# Cancer Patients’ Age-Related Benefits from Mobile Neurofeedback-Therapy in Quality of Life and Self-efficacy: A Clinical Waitlist Control Study

**DOI:** 10.1007/s10484-022-09571-1

**Published:** 2022-11-18

**Authors:** Kira Schmidt, Marvin Krawutschke, Axel Kowalski, Saskia Pasche, Anna Bialek, Theresa Schweig, Benjamin Weismüller, Mitra Tewes, Martin Schuler, Rainer Hamacher, Bernhard W. Müller, Dirk Schadendorf, Eva-Maria Skoda, Martin Teufel, Madeleine Fink

**Affiliations:** 1 Clinic for Psychosomatic Medicine and Psychotherapy, LVR University Hospital Essen, University of Duisburg-Essen, Virchowstraße 174, 45147 Essen, Germany; 2 Center for Translational Neuro- and Behavioral Sciences (C-TNBS), University of Duisburg-Essen, 45147 Essen, Germany; 3 NeuroFit GmbH, Krefeld, Germany; 4 IB University of Applied Health and Social Sciences, Berlin, Germany; 5 Department of Medical Oncology, West German Cancer Center, University Hospital Essen, University of Duisburg-Essen, Essen, Germany; 6 German Cancer Consortium (DKTK), Partner Site University Hospital Essen, and German Cancer Research Center (DKFZ), Essen, Germany; 7 Department of Psychiatry and Psychotherapy, LVR University Hospital Essen, Medical Faculty, University of Duisburg-Essen, Essen, Germany; 8 Department of Psychology, University of Wuppertal, Wuppertal, Germany; 9 Clinic for Dermatology, University Hospital Essen, Essen, Germany

**Keywords:** Neurofeedback, Age effect, Cancer, EORTC QLQ-C30, Quality of life, Self-efficacy

## Abstract

Electroencephalographic neurofeedback (EEG NF) can improve quality of life (QoL) and reduce distress by modifying the amplitude of selected brain frequencies. This study aims to investigate the effects of NF therapy on QoL and self-efficacy in cancer patients and to explore age-related reactions. In a waitlist control paradigm, psychometric data (*EORTC QLQ-C30, General Self-Efficacy Scale*) of 20 patients were collected at three different time points, each five weeks apart. An outpatient 10-session NF intervention (mobile) was conducted between the second and third measurement point. QoL and self-efficacy changed significantly over time (QoL: *F*(2,36) = 5.294, *p* < .05, *η*^2^ = .227; Self-efficacy: *F*(2,26) = 8.178, *p* < .05, *η*^2^ = .386). While QoL increased in younger patients, older patients initially showed a decrease in QoL, which then increased during intervention. Younger patients did not differ from older patients in QoL in both waitlist control (T0-T1) and intervention phase (T1–T2). QoL in older patients significantly differed between waitlist control and intervention phase (*Z* = − 2.023, *p* < .05, *d* = 1.085). Self-efficacy increased in both age categories. Younger and older patients did not differ in self-efficacy in waitlist control, but in intervention phase (*F*(1,16) = 7.014, *p* < .05, *η*^2^ = .319). The current findings suggest that NF therapy is a promising treatment modality for improving QoL in cancer patients. Our study reveals NF being a tool to influence self-efficacy, which should receive more appreciation in clinical care. However, the effect of NF in different age groups as well as the influence on further cancer-related symptoms should be investigated in future research.

**Supplementary Information:**

The online version contains supplementary material available at 10.1007/s10484-022-09571-1.

## Background

In cancer patients, quality of life (QoL) is often impaired by various factors. Even years after a cancer diagnosis, women under 40 years of age continue to face problems that women without breast cancer do not face (Kroenke et al., 2004). This includes a decline in physiological roles, social functioning, mental health, and increase of physical pain. Managing the psychological distress of this patient population is an essential component of clinical oncology (Brown et al., 2005). QoL is not only influenced by the severe challenges patients have to face but also since psychological well-being represents an important contributor to the expression of QoL (Kang et al., 2017). Although in recent years, QoL and mental health impairments were thought to be mainly influenced by clinical and pathological salient factors, psychological resources, such as self-efficacy, were also found to predict QoL (Kostka & Jachimowicz, 2010). Self-efficacy is defined as one's belief in their ability to achieve desired results through their own actions using their own skills and abilities (Bandura et al., 1999). Cancer patients display varying levels of self-efficacy after treatment (Foster et al., 2015) and previous studies demonstrated a relationship between self-efficacy and QoL (Baik et al., 2020; Kiaei et al., 2016). Not only was higher self-efficacy related to higher QoL but also to increased well-being in social, emotional, and functional domains (Baik et al., 2020). In addition, higher self-efficacy resulted in lower burden of cancer-related symptoms and less cancer-specific stress. Thus, QoL and self-efficacy are important constructs in the treatment of patients in the medical context. Moreover, it constitutes an eminent measure for patients and clinicians in the process of decision making about an appropriate treatment (Alghamedi et al., 2018). Additionally, higher QoL before cancer treatment was associated with higher survival (DiSipio et al., 2011; Kidane et al., 2017). Interestingly, several studies found age-related differences in QoL of cancer patients with younger patients presenting lower levels than older patients (Hopwood et al., 2007; Sammarco, 2009; Wenzel et al., 1999). To the best of our knowledge, research did not yet address the expression of self-efficacy in different age groups of cancer patients.

The number of cancer patients persistently rose in recent years and cancer represents the second leading cause of death worldwide with approximately 9.9 million deaths in 2020 (Sung et al., 2021). Since the 70s, the number of patients in Germany nearly doubled, while the mortality rate continued to decline (Barnes et al., 2016). This leads to an even larger number of cancer survivors whose psycho-oncological follow-up needs to be covered clinically. Nevertheless, literature is lacking on potentially helpful therapies improving QoL. However, evidence from biofeedback and neurofeedback (NF) studies demonstrated positive effects on QoL and e.g. short-term memory (Elahi Nejad et al., 2020; Sarvghadi et al., 2019). Electroencephalographic neurofeedback (EEG NF) represents an evidence based procedure of behavior therapy and innovative complementary therapies (Luctkar-Flude & Groll, 2015). EEG NF is a non-invasive brain training allowing real-time processing of EEG signals, extraction of the parameters of interest, and subsequent visual or auditory feedback representation (Hetkamp et al., 2019; Micoulaud-Franchi et al., 2015). Behavior modification can be achieved by modulating brain activity, e.g. through volitional control (Micoulaud-Franchi et al., 2015). Markiewcz (2017) showed that NF also facilitates synaptogenesis and can reconnect neuronal circuits and generate new ones by long-term potentiation (Markiewcz, 2017). By conditioning, selected frequency bands’ amplitude is altered, leading to the training effect (Luctkar-Flude & Groll, 2015). However, research indicates that the rapid response of the brain to the performed training is not solely due to conditioning responses (Othmer & Othmer, 2016). Example responses to this represent rapid and unexpected state changes and symptom relief. Thus, the brain seems to use and derive additional information from the training signal. The largest application fields of NF include attention and hyperactivity disorders, affective disorders, stroke, epilepsy, migraine, and chronic insomnia (Micoulaud-Franchi et al., 2015).

Based on the categorization of NF approaches by Luctkar-Flude and Groll (2015) the targeted mechanism-based, symptom-responsive approach is mostly used in the treatment of psychopathologies such as anxiety, depression, and pain syndromes with alpha and theta/beta as the selected brainwave targets. While occipital alpha activity is suggested as a surrogate marker for relaxing state (Niedermeyer, 1997), central frontal theta and beta activity are associated with arousal (Haenschel et al., 2000; Strijkstra et al., 2003). Hence, alpha-NF-training aims to increase alpha-frequency in order to evoke a relaxed brain state, whereas theta/beta-NF-training aims to decrease theta/beta-frequency in order to reduce arousal. Thus, alpha- and theta/beta-NF are often used and have been shown to be effective in treating symptoms as fatigue, depression, and anxiety, which are also common in cancer patients (Hetkamp et al., 2019) and whose decrease we assume being associated with an increase of QoL. In the traditional unipolar mode of NF an active electrode is placed on the skull close to relevant cerebral regions and a reference electrode is placed in the vestibular region (Marzbani, Marateb & Mansourian, 2016). However, in most NF protocols the active electrode is placed at Cz (Haus et al., 2013), measuring activity in the somatosensory cortex, including attention, mental processing, calmness, emotion and empathy (Marzbani et al., 2016). Moreover, the Cz receives projections of the thalamic structures, which are associated with memory processes and executive functions of attention and information processing (Fama & Sullivan, 2015). In addition, the location on Cz has the benefit that mobile NF is easy to conduct and can also be performed independently by patients in the home setting.

Despite its proven efficacy in other diseases, NF as an integrative psycho-oncological treatment for cancer patients is very rarely used (see Hetkamp et al., 2019). Cancer patients often suffer from pain, fatigue, anxiety, depression, and cognitive impairment (Luctkar-Flude & Groll, 2015). However, research suggests that NF-therapy can alleviate many long-term symptoms and improve QoL of cancer patients (Luctkar-Flude & Groll, 2015; Sarvghadi et al., 2019). A recent review strongly corroborates the effectiveness of NF, even despite a small number of studies (Hetkamp et al., 2019). Most studies included in the meta-analysis reduced cancer-related symptoms. Thus, improvements in QoL, pain symptoms, fatigue, and cognitive impairment were demonstrated. Moreover, an Iranian pilot study examined 20 breast cancer patients receiving either radio- or chemotherapy in a randomized experimental study design (Sarvghadi et al., 2019). Patients in the intervention group received 20 NF sessions over a period of four weeks and demonstrated both improved short-term memory and increased QoL at the end of the intervention. However, to our knowledge there are to date no research findings about the action mode of NF in different age groups. Therefore, the aim of the present waitlist-controlled treatment study was to investigate the influence of NF on QoL and self-efficacy in different age groups of cancer patients. Based on current research, we hypothesized that both QoL and self-efficacy would increase with NF therapy. Moreover, we expected to detect a lower QoL in younger than in older patients.

## Method

This work was a prospective, controlled and clinical study with a waiting list, designed as part of a larger project (registration in the German Register of Clinical Studies: DRKS00015773). The study was approved by the Ethics Committee of the Medical Faculty of the University of Duisburg-Essen (No. 18-8079-BO).

We followed the *consensus on the reporting and experimental design of clinical and cognitive-behavioral neurofeedback studies* (CRED-nf checklist) in conducting and reporting this clinical trial (Ros et al., 2020).

### Procedure and Participants

Data collection was conducted from October 25, 2018 to April 22, 2021. Due to the COVID-19 lockdown, a total interruption of the project had to take place, and recruitment stopped from March 30, 2020 to June 1st, 2020, and from December 2020 to March 15, 2021. Participants were recruited at the West German Cancer Center (German: Westdeutsches-Tumor-Zentrum, WTZ), the comprehensive cancer center of the University Hospital Essen, via social media, and common local newspapers. Inclusion criteria were diagnosis of malignant oncologic disease according to the Union for International Cancer Control (UICC), available informed consent, and age between 18 and 75 years. Patients with major depression or acute suicidality, psychotic symptoms/disease, alcohol/drug abuse, and/or central nervous disease (cerebral metastases), or poor German language skills were excluded. At study entry (time point T0), initial self-report questionnaires were completed. After a waiting period of five weeks, participants completed an additional self-report questionnaire (time point T1) and started NF therapy conducted for another five-week period. Self-assessment questionnaires were collected again after the intervention (time point T2). For ethical reasons, all patients received an intervention after the waiting phase. Therefore, all data gathered from T0 to T1 were considered as waitlist control phase and those gathered from T1 to T2 as intervention phase.

### Neurofeedback Therapy

**Fig. 1:**
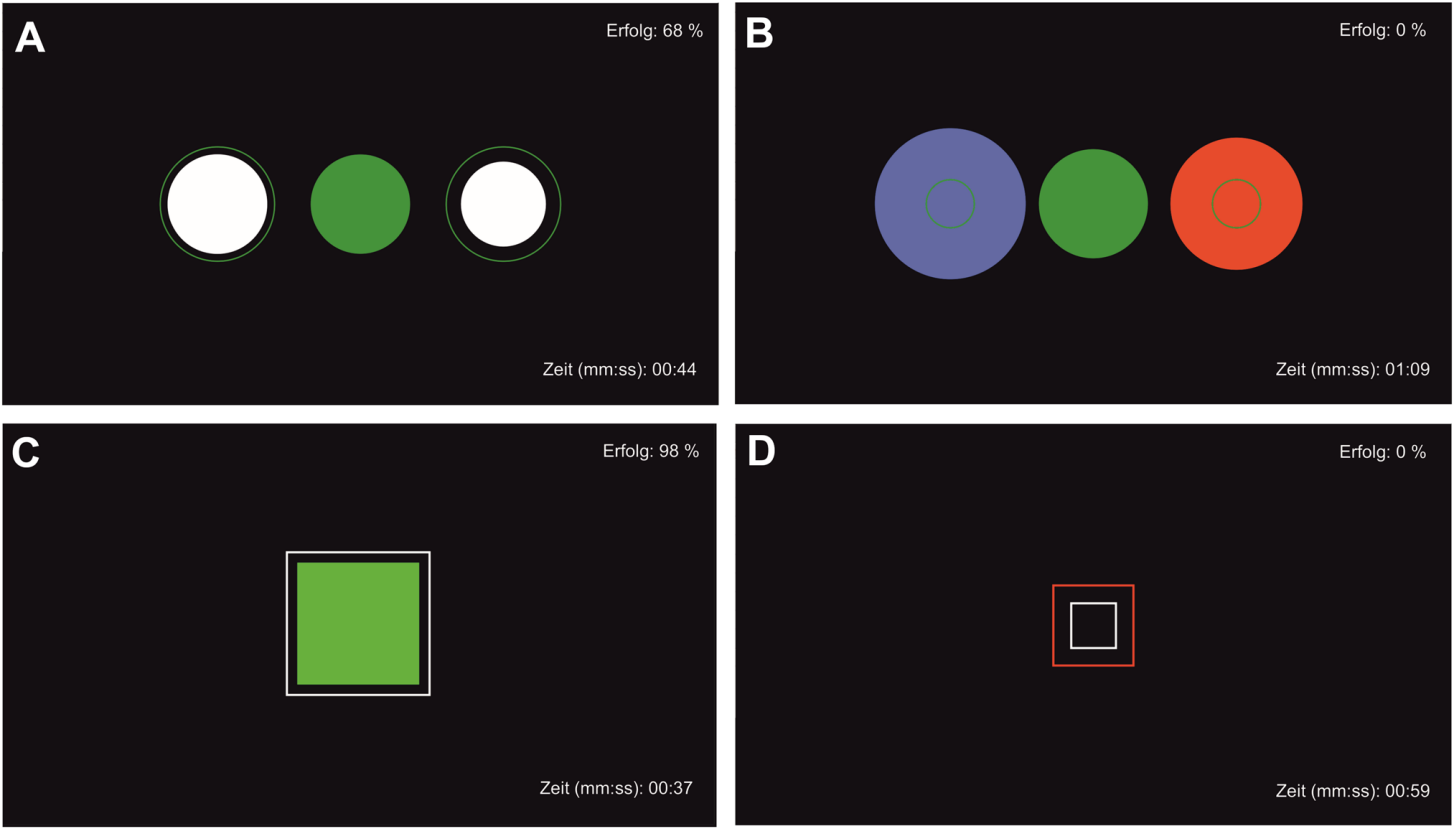
Stimuli of neurofeedback therapy. The success rate (“Erfolg”) is displayed in the upper right corner, the elapsed time during the exercise in the lower right corner. **A** target state relaxation, **B** target state relaxation not reached, **C** target state attention, **D** target state attention not reached

The mobile NF therapy was performed using a modified *Mind Wave* headset (NeuroSky & Inc., 2011) and positioning the electrode at coordinate Cz. The *BioEra Pro* software was used to transmit and process the recorded signal from the headset to the computer in order to visualize it. Using *Fast Fourier Transformation based filter algorithms*, the raw signals were decomposed into individual frequency bands. This output signal was used as feedback displayed on the monitor as the target condition during training. As stimuli, subjects saw geometric figures, which changed depending on the degree of match with the target condition (Fig. 1). Stimuli were either circles or squares changing their colors from green and white to blue and red indicating high or low accordance, respectively, to the target condition. The rate of success was displayed in the upper right corner and elapsed time during the exercise in the lower right corner of the screen. The investigator (MF) was present throughout the training, but did not provide verbal feedback or performed any manipulation of the feedback process. The NF intervention included a minimum of six and a maximum of ten training sessions, each lasting 40–45 min. The sessions took place in the outpatient clinic, twice a week over a period of five weeks and followed the following structure: resting state for approximately five min, alpha training (9–13 Hz attenuation) and reduction of theta/beta (> 20 Hz) for ten min, resting state for approximately five min, target theta/beta ratio ≤ 2.5 for approximately five min, resting state for approximately five min, alpha training (9–13 Hz attenuation) and reduction of theta/beta (> 20 Hz) for ten min (based on Hetkamp et al., 2019).

For descriptive statistics of quantitative data of the first and sixth NF sessions, see supplements Table 1. Wilcoxon signed rank tests revealed a significant difference between alpha frequency bands of first and sixth NF session (alpha 1: *Z* =  − 2.158, *p* < 0.05; alpha 2: Z =  − 2.534; *p* < 0.05; see supplements Table 2). No significant differences between theta/beta waves of first and sixth NF session were found. Change in alpha and theta/beta frequency bands did not correlate with psychometric data (QoL, self-efficacy) for all participants in total as well as for over and under 55-year-old patients (see supplements table 3). However, changes in self-efficacy correlated with the change in alpha frequency bands of the first alpha training sections from the first to the sixth NF session in under 55-year-old participants (*r* = -0.747, *p* < 0.05).

**Table 1:** Demographic data of the participants

	*n*	Percentage (%)
*Gender*		
Female	13	65
Male	7	35
*Living situation*		
With partner	16	80
Alone	4	20
*Children*		
Yes	13	65
No	7	35
*Employment status*		
Employed	10	50
On sick leave	5	25
Retired	3	15
Other	2	10
*Education*		
High school diploma	10	50
Secondary school degree („*Realschule* “)	6	30
Secondary school degree („*Hauptschule* “)	2	10
Missing	2	10
*Cancer type*		
Breast	5	25
Melanoma	4	20
Lung	3	15
Pancreas	2	10
Angiosarcoma	1	5
Endometrial	1	5
Multiple myeloma	1	5
Lymphoma	1	5
Non-hodgkin lymphoma	1	5
Seminoma	1	5
*Tumor stage (UICC)*		
I	3	15
II	1	5
III	8	40
IV	8	40

*N* = 20

### Measurement Instruments

The questionnaire consisted of sociodemographic items as well as validated instruments. Sociodemographic data were assessed including age, gender, living situation, education, and employment status. Moreover, cancer type and tumor-stage were assessed. QoL was measured with the *European Organization of Research and Treatment of Cancer-related Quality of Life* (EORTC QLQ-C30) questionnaire, which is a multidimensional self-report instrument and includes 30 items on nine different scales (Aaronson et al., 1993). Items were answered on a 4-point scale ranging from 1 = "not at all" to 4 = "very much". It has acceptable reliability (Cronbach's α > 0.70) and validity (Aaronson et al., 1993). Self-efficacy was measured using the *General Self-Efficacy Scale* (GSE), which is a self-report measure of general optimistic self-beliefs using 10 items (Schwarzer, 1999). Item responses were given on a 4-point scale ranging from 0 = "not true" to 4 = "true exactly." The internal consistency of this measurement instrument is good (Cronbach's α = 0.80 to 0.90).

### Statistical Analysis

Statistical analyses were performed using the Statistical Program for Social Sciences SPSS version 26 (IBM, New York). Figures were created using CorelDRAW X5 (Corel, Ottawa) and Prism 9.0.2 (GraphPad, San Diego). For all analyses, the significance level was set at α = 0.05. All analyses were conducted after outlier-correction (1 *SD*) via boxplots. In the case of non-normally distributed data, Spearman correlations were calculated. Single-factor analyses of variance (ANOVA) were conducted even in case of non-normally distributed data, since those are considered to be robust (Wilcox, 2011). ANOVAs with repeated measures were calculated for the EORTC QLQ-C30 and the GSE, controlled for patient age, to examine the change in symptomatology over time. If sphericity was violated, the Greenhouse–Geisser correction was used. For analyses of age effects, a median split (< 55 and > 55 years of age), ANOVAs, and Wilcoxon signed rank tests were used. Effect sizes were either defined with Cohen’s *d* or eta squared (η^2^). Moreover, we calculated correlations and constructed a regression model with age and self-efficacy predicting QoL after NF therapy. Additionally, mean values of EEG-frequency bands (Hz) during the first six NF sessions were calculated over the mean 60 s of each training session. Wilcoxon signed rank tests were calculated to compare the frequency bands of the first and sixth NF session.

## Results

### Sample Characteristics

A total of 23 cancer patients participated, three of whom discontinued due to the COVID-19 pandemic. This resulted in a sample of 20 patients (13 female, 7 male) with a mean age of 52.80 (SD = 10.768, median = 54.50) years (range 31–73). Table 1 shows the demographic information. Subjects had various cancer types with average WHO stages of III and IV. Each patient completed at least six NF sessions, with an average of 8.45 sessions attended (range 6–10). Number of sessions did not differ significantly between under and over 55-year-old patients (*Z* =  − 1.812, *p* = 0.07).

### Quality of Life

Table 2 displays the descriptive statistics. When controlled for age, the ANOVA presented a significant change of QoL over time (*F*(2,36) = 5.294, *p* < 0.05, *η*^2^ = 0.227). Patient Age interacted significantly with QoL at measured time points (*F*(2,36) = 5.284, *p* < 0.05, *η*^2^ = 0.227). Since no significant changes in QoL emerged from pairwise comparisons using Bonferroni correction, it was not possible to detect at which time points QoL differed.

**Table 2:** Descriptive statistics of quality of life by EORTC QLQ-C30 and self-efficacy by GSE at all three measurement time points T0, T1, and T2 (upper part), and regression model with age and self-efficacy predicting QoL (lower part)

Outcome	*N*	*M*	*SD (SE)*	*S (SE)*	*K (SE)*
EORTC T0	20	60.00	22.330 (4.993)	− 0.847 (0.512)	− 0.657 (0.992)
EORTC T1	20	57.90	23.633 (5.284)	− 0.522 (0.512)	− 0.520 (0.992)
EORTC T2	20	63.35	22.843 (5.108)	− 0.167 (0.512)	− 1.070 (0.992)
GSE T0	18	28.28	3.997 (0.942)	− 0.752 (0.536)	1.831 (1.038)
GSE T1	16	29.44	2.502 (0.626)	0.021 (0.564)	1.312 (1.091)
GSE T2	17	29.65	2.548 (0.618)	− 0.708 (0.550)	1.836 (1.063)

Predictor	*β*	*βse*	*t*	*p*
Intercept	− 26.104	19.530	− 1.337	0.203
GSE_intervention_	3.951	1.545	2.558	≤ 0.05
Age	0.650	0.351	1.850	0.086

Total *R*^2^ = 0.319 (*F*(2) = 3.278; *p* = 0.068; *N* = 16). All analyses were conducted outlier-corrected

*EORTC* European Organization for Research and Treatment of Cancer-Related Quality of Life (EORTC QLQ-C30), *GSE* General Self-Efficacy Scale

**Fig. 2:**
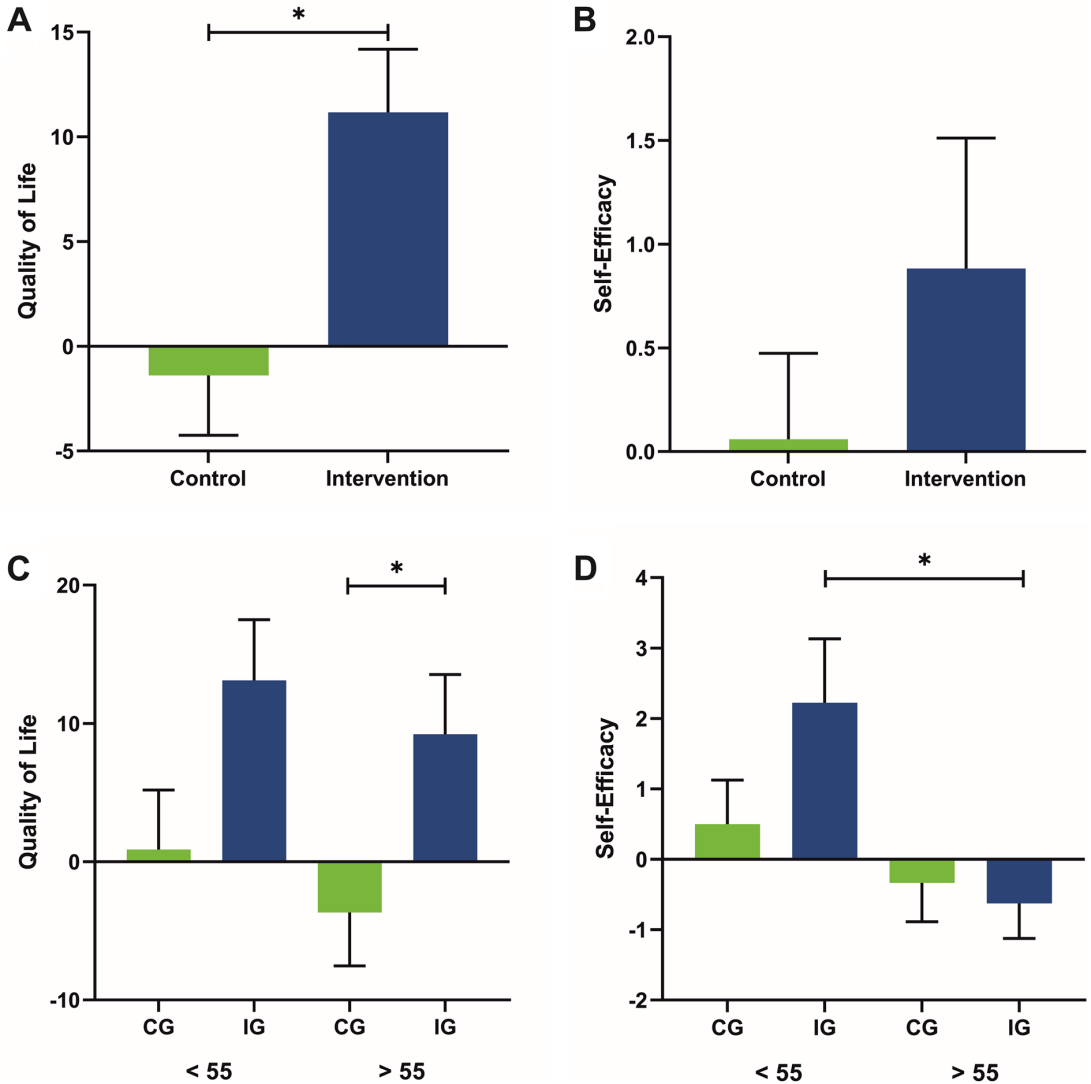
**A** Quality of life (QoL) in waitlist control (*M* =  − 1.389, *SE* = 2.857) and intervention phase (*M* = 11.167, *SE* = 3.019). **B** Self-efficacy in waitlist control (*M* = 0.059, *SE* = 0.415) and intervention phase (*M* = 0.882, *SE* = 0.629). **C** QoL of patients under 55 years of age in waitlist control (CG; *M* = 0.889, *SE* = 4.289) and intervention phase (IG; *M* = 13.111, *SE* = 4.389) and of over 55 year-old patients in waitlist control (*M* =  − 3.667, *SE* = 3.873) and intervention phase (*M* = 9.222, *SE* = 4.307). **D** Self-efficacy of patients under 55 years of age in waitlist control (*M* = 0.50, *SE* = 0.627) and intervention phase (*M* = 2.222, *SE* = 0.909) and of over 55 year-old patients in waitlist control (*M* = − 0.333, *SE* = 0.552) and intervention phase (*M* = − 0.625, *SE* = 0.498). Error bars show the standard errors (*SE*). *p < 0.05

Age did not correlate with the change in QoL from T1 to T2 (*r* =− 0.114, *p* = 0.653). Descriptively, the ANOVA showed an increase of QoL in younger patients over the course of the measurements (T0: *M* = 57.50, *SD* = 23.680; T1: *M* = 62.50, *SD* = 17.784; T2: *M* = 70.90, *SD* = 21.707; *F* = 2.655, *p* = 0.098), whereas the QoL of older patients initially decreased and then increased from T1 to T2 (T0: *M* = 62.50, *SD* = 21.864; T1: *M* = 53.30, *SD* = 28.562; T2: *M* = 55.80, *SD* = 22.444; *F* = 1.078, *p* = 0.361). Disease severity showed no association with age (*r* = 0.348, *p* = 0.133). QoL of our total sample differed significantly between waitlist control and intervention phase (*Z* =  − 2.717, *p* < 0.05, *d* = 1.016; Fig. 2A). Non-parametric group comparisons did not reveal any significant difference in QoL between under (*M* = 0.889, *SE* = 4.29) and over (*M* =− 3.667, *SE* = 3.873) 55-year-old patients in the waitlist control phase (∆T1–T0; *F*(1,17) = 0.621, *p* = 0.442, *η*^2^ = 0.037; Fig. 2C) and between under (*M* = 13.111, *SE* = 4.389) and over (*M* = 9.222, *SE* = 4.307) 55-year-old patients in the intervention phase (∆T2-T1; *F*(1,17) = 0.400, *p* = 0.536, *η*^2^ = 0.024). Wilcoxon signed rank tests revealed a significant difference between waitlist control and intervention phase of older patients (*Z* =  − 2.023, *p* < 0.05, *d* = 1.085; Fig. 2C). No differences were found in QoL of younger patients between waitlist control and intervention phase (*Z* =  − 1.873, *p* = 0.061, *d* = 0.984).

### Self-efficacy

Controlled for age, the ANOVA showed a significant change in self-efficacy across the three time points (*F*(2,26) = 8.178, *p* < 0.05, *η*^2^ = 0.386). Patient age interacted significantly with self-efficacy at all three measurement points (*F*(2,26) = 7.840, *p* < 0.05, *η*^2^ = 0.376). Since pairwise comparisons with Bonferroni correction showed no significant changes, it was not possible to detect at which time points self-efficacy differed.

No correlation of self-efficacy and age for ∆T2-T1 (*r* = − 0.429, *p* = 0.086) was found. Descriptively, the ANOVA showed an increase of self-efficacy in under 55-year-old patients from time point T0 (*M* = 27.75, *SD* = 2.915) to T1 (*M* = 29.38, *SD* = 1.847) and T2 (*M* = 30.29, *SD* = 1.113; *F* = 2.378, *p* = 0.135). For older patients, self-efficacy also increased from T0 (*M* = 28.70, *SD* = 4.809) to T1 (*M* = 29.50, *SD* = 3.162) and T2 (*M* = 29.20, *SD* = 3.190; *F* = 1.538, *p* = 0.249). Self-efficacy of our total sample did not differ significantly between waitlist control and intervention phase (*Z* = − 0.714, *p* = 0.475, *d* = 0.247; Fig. 2B). Non-parametric group comparisons did not reveal any significant difference in self-efficacy between under (*M* = 0.500, *SE* = 0.627) and over (*M* = − 0.333, *SE* = 0.553) 55-year-old patients in the waitlist control phase (∆T1–T0; *F*(1,16) = 1.003, *p* = 0.333, *η*^2^ = 0.063; Fig. 2D). Self-efficacy between under (*M* = 2.222, *SE* = 0.909) and over (*M* = − 0.625, *SE* = 0.498) 55-year-old patients differed significantly in the intervention phase (∆T2-T1; *F*(1,16) = 7.014, *p* < 0.05, *η*^2^ = 0.319). Wilcoxon signed rank tests revealed no significant differences in self-efficacy between waitlist control and intervention phase of younger (*Z* =  − 1.156, *p* = 0.248, *d* = 0.584) and older (*Z* = − 0.271, *p* = 0.786, *d* = 0.132) patients.

### Correlations and Regression

QoL and self-efficacy at time point T0 did not correlate (*r* = 0.349, *p* = 0.078). Self-efficacy and QoL correlated significantly in the intervention (*r* = 0.463, *p* < 0.05), but not in the waitlist control phase (*r* = − 0.274, *p* = 0.152). Although not significant, the regression model shows a trend (*p* = 0.068), according to which self-efficacy in the intervention phase predicted QoL (Table 2).The model provides an explained variance of 31.9%. Age did not predict QoL in the intervention.

## Discussion

Since to date, the number of studies is limited, the aim of the current waitlist-controlled treatment study was to investigate the impact of NF therapy on QoL and self-efficacy in different age groups of cancer patients and survivors and its interactions. The results show a significant improvement in health-related QoL and self-efficacy over time when controlled for patient age. QoL differed significantly between waitlist control and intervention phase indicating NF therapy had a positive effect on QoL. These findings are consistent with further literature (Sarvghadi et al., 2019).

As basis for further analyses, the non-parametric group comparisons revealed a significant change of alpha frequency bands after NF therapy. We assume an association between the change of frequency bands and improvements of QoL and self-efficacy based on previous literature (e.g. Elahi Nejad et al., 2020; Prinsloo et al., 2018; Sarvghadi et al., 2019; Teufel et al., 2013). Our study reveals a decrease of affective cancer-related symptoms, which is in association with an increase of alpha frequency bands. This underlines previous literature, which suggest symptoms of cancer patients being based on changes in cortical activity (Prinsloo et al., 2014, 2017).

In order to examine age effects, we divided our cohort into two groups by using the median split. We chose this method to form subgroups of equal size. When investigating different age groups of cancer patients, various factors, e.g. tumor stage or cancer type, can have an influence on the general state of patients and need to be taken into consideration. Therefore, health condition of various patients, including aspects of QoL and/or self-efficacy, might be diverse regardless of the patients’ age but depending on cancer type and/or tumor stage. However, the focus of this study was to examine psychometrics in order to generalize the results to a broad clientele, which constitutes a key characteristic of tumor centers. The results show an age effect, in which QoL increased more in younger people during the waitlist period than in older patients. However, changes in QoL due to the intervention are higher for older patients. Previous studies confirmed the finding of lower QoL in younger patients. A comparison of over- and under-55-year-old breast cancer patients demonstrated that younger patients had significantly lower QoL (Wenzel et al., 1999). Lower scores in socioeconomic and psychological/spiritual QoL (Sammarco, 2009) and in physical and social functioning and financial difficulties were also observed in younger compared to older patients (Hopwood et al., 2007). In contrast, older age was associated with better social but poorer physical function (Hopwood et al., 2007) suggesting older age being associated with greater severity of illness. However, previous studies showed that younger cancer patients experience higher emotional distress from the disease than older patients (Cordova et al., 1995; Mor et al., 1994), especially regarding social and family aspects (Dunn & Steginga, 2000; Mor et al., 1994). Younger patients are at greater risk for long-term QoL problems since they exhibit more depressive symptoms, fatigue, poorer attentional function, and sexual dysfunction (Champion et al., 2014). Furthermore, younger patients showed greater personal growth (Champion et al., 2014). This may explain the greater improvement of QoL in younger than in older patients of our sample. Younger patients were found to show greater difficulties in coping with side effects of cancer treatment, regulating their affect, and maintaining a positive baseline attitude, which may also explain their lower QoL in the beginning of the study (Merluzzi & Martinez Sanchez, 1997). The NF therapy in this study may have broadened the patients' coping assortment, providing another opportunity to actively deal with disease-related stressors. Older patients may have more coping strategies at their disposal beforehand, due to which NF therapy had a less pronounced effect. The results also indicate that older patients initially showed a deterioration in QoL, which then increased again with the start of NF therapy. Moreover, non-parametric group comparisons revealed that older patients significantly differed in QoL between waitlist control and intervention phase. This observation is consistent with other studies, which also found a decrease in QoL with increasing age (Arndt et al., 2004; Bottomley, 2002), and underlines the importance of an intervention in this age group.

Self-efficacy steadily increased descriptively over the measurement, with less improvement during the NF intervention. Furthermore, an age effect displayed a stronger change in self-efficacy in younger than in older patients. The non-parametric group comparisons show a significant difference in self-efficacy between younger and older patients during the intervention. Based on the definition of self-efficacy, study participation may give patients a sense of being able to independently control their situation and treat their cancer-related symptoms. In this context, the feeling of control may be mediated by the expression of coping strategies, of which younger patients acquired fewer than older patients (Wenzel et al., 1999). Older patients may thus have perceived higher control over their situation from the beginning of the disease due to their larger repertoire of coping strategies and thus experienced higher self-efficacy in dealing with their cancer and associated symptoms. Therefore, at baseline, older patients showed higher self-efficacy than younger patients. The younger subjects' sense of control increased by participating in the study, even though no intervention had yet occurred. Interestingly, the present study reveals a negative correlation between self-efficacy and alpha frequency, i.e. alpha frequency of the first alpha training during session one to six, in under 55-year-old participants. This implies that younger participants might have experienced themselves as less self-efficacious during the first alpha training sections. Due to the lack of literature about the relationship between alpha frequency and self-efficacy, it is necessary to further investigate this finding in future research.

Since self-efficacy and QoL significantly correlated in the intervention, but not in the waitlist control phase, self-efficacy can be assumed as an important predictor for the QoL treatment effect. Other studies also showed that self-efficacy positively influences QoL (Chirico et al., 2017; Rottmann et al., 2010). This emphasizes the importance of mediating self-efficacy skills in order to increase the QoL of cancer patients.

### Study Limitations

The fact that above all the regression analysis did not reveal significant effects might be based on the small sample size of 20 patients. A replication study with a larger sample would potentially provide clearer results and should be conducted in the future. In addition, the sample of the present study displayed heterogeneity regarding cancer type and homogeneity regarding tumor stage with most patients having a tumor stage of either III or IV and therefore suffering from a severe degree of the cancer disease. This prevents drawing conclusions about the effects of NF therapy on a particular cancer type or tumor stage. Even though our results show positive effects associated with the conducted NF therapy, future studies should investigate the effect of NF therapy in different age groups with respect to cancer type and tumor stage. Another limitation of this study refers to the relationship between NF therapy and improvements in QoL and self-efficacy. Unfortunately, we did not find a significant correlation between change in alpha frequency and improvement in QoL as a highly global measure, but based this conclusion on existing research. Additionally, due to the lack of a control group, the change in psychometric data might be influenced by a placebo effect. Therefore, future studies should include a control group or examine a larger sample of patients. However, since our study is based on a small sample, the findings can be interpreted as first indications, which need to be investigated in larger studies. Moreover, only a minimum number of six NF therapy sessions and a maximum number of ten sessions were established, whereas prior studies conducted NF therapy with a higher number of sessions ranging from 10 to 30 (Alvarez et al., 2013; Prinsloo et al., 2018; Sarvghadi et al., 2019). This suggests that the number of NF sessions performed here could be too low to produce clear effects on symptomatology. Moreover, the used EEG hardware, i.e. Neurosky MindWave headset, might be insufficient to precisely measure brain frequency bands in a scientific context due to insensitive electrodes and signal transmission quality. However, since the Neurosky MindWave headset might be insufficient for scientific investigation of brain frequency bands, we focused on psychometric data and were able to test efficacy of NF therapy as a clinical-operational therapy method in a representative cohort. Therefore, this device offers a good opportunity for patients to easily perform a mobile NF training in their home context or during somatic therapy in order to improve QoL and self-efficacy.

Another limitation of this study refers to the possibility of access to NF training. Unfortunately, not every patient has access to psycho-oncological support services. Moreover, NF is not implemented in every clinic and not every therapist has the necessary knowledge and technical devices in order to offer NF therapy.

### Clinical Implications

Our study underlines the effectiveness of NF therapy, which constitutes an easy-to-use, low-cost paradigm. The mobile NF training can be conducted without a therapist and can be used by patients of any cancer stage. Due to its mobility, it can offer a great opportunity for outpatients as well as inpatients to influence their QoL and self-efficacy and can provide support in several life situations.

To date, there are no other research findings regarding age effects of NF therapy in cancer patients. The results of this study suggest that NF intervention may be more effective in younger than in older patients. Accordingly, NF therapy may be predominantly applicable in young patients. However, the present results also show that the QoL of older patients deteriorates without intervention. Therefore, especially this age group should find consideration in clinical care. Self-efficacy additionally constitutes a significant predictor of QoL, which might be influenced by available coping strategies. Accordingly, self-efficacy and coping strategies should receive more attention in psycho-educational settings. Future research should further investigate potential long-term effects of NF therapy in cancer patients. Additionally, it might be important to investigate age-related differences in baseline EEG measures during NF therapy.

## Conclusion

The increasing number of oncological patients in Germany leads to a higher demand for psycho-oncological aftercare. Especially QoL represents an important aspect, since cancer patients are confronted with a particularly large number of challenges. This study was able to demonstrate the use of NF therapy as a possible treatment option for cancer patients. In addition to an improvement in QoL, NF therapy also represents a tool to regain a feeling of control, which is usually lower in cancer patients. Nevertheless, the results show a promising impact of NF therapy on QoL and self-efficacy of cancer patients, which should receive more attention in clinical care.

## Supplementary Information

Below is the link to the electronic supplementary material.

Supplementary file1 (DOCX 23 kb)

## Data Availability

All data are available on request from the responsible author.
