# Supplementary material for: Cancer Patients’ Age-Related Benefits from Mobile Neurofeedback-Therapy in Quality of Life and Self-efficacy: A Clinical Waitlist Control Study
Source: Appl Psychophysiol Biofeedback. 2022 Nov 18;48(2):217–27. doi: 10.1007/s10484-022-09571-1 (PMC9676905; doi:10.1007/s10484-022-09571-1)
Supplement: Supplementary file 1 — Supplementary file1 (DOCX 23 kb) [file 10484_2022_9571_MOESM1_ESM.docx]

Supplements

corresponding to the article

**Cancer Patients’ Age-related Benefits from Mobile Neurofeedback-Therapy in Quality of Life and Self-Efficacy: A Clinical Waitlist Control Study.**

**Kira Schmidt*** (kira.schmidt@uni-due.de)^1,2^, Marvin Krawutschke (marvin.krawutschke@stud.uni-due.de)^1,2^, Axel Kowalski (axel.kowalski@neurofit-akademie.de)^3,4^, Saskia Pasche (saskia.pasche@lvr.de)^1,2^, Anna Bialek (anna.bialek@lvr.de)^1,2^, Theresa Schweig (theresa.schweig@stud.uni-due.de)^1,2^, Benjamin Weismüller (benjamin.weismueller@hhu.de)^1,2^, Mitra Tewes (mitra.tewes@uk-essen.de)^5,6^, Martin Schuler (martin.schuler@uk-essen.de)^5,6^, Rainer Hamacher (rainer.hamacher@uk-essen.de)^5,6^, Bernhard W. Müller (bernhard.mueller@uni-due.de)^7,8^, Dirk Schadendorf (dirk.schadendorf@uk-essen.de)^6,9^, Eva-Maria Skoda (eva-maria.skoda@uni-due.de)^1,2^, Martin Teufel (martin.teufel@uni-due.de)^1,2^, Madeleine Fink (madeleine.fink@uni-due.de)^1,2^

^1^ Clinic for Psychosomatic Medicine and Psychotherapy, LVR University Hospital Essen, University of Duisburg-Essen, Essen, Germany

^2^ Center for Translational Neuro- and Behavioral Sciences (C-TNBS), University of Duisburg-Essen,

45147 Essen, Germany

^3^ NeuroFit GmbH, Krefeld, Germany

^4^ IB University of Applied Health and Social Sciences, Berlin, Germany

^5^ West German Cancer Center, Department of Medical Oncology, University Hospital Essen, University of Duisburg-Essen, Essen Germany

^6^ German Cancer Consortium (DKTK), Partner Site University Hospital Essen, and German Cancer Research Center (DKFZ), Essen, Germany

^7^ Department of Psychiatry and Psychotherapy, LVR University Hospital Essen, Medical Faculty, University of Duisburg-Essen, Essen, Germany

^8^ Department of Psychology, University of Wuppertal, Wuppertal, Germany

^9^ Clinic for Dermatology, University Hospital Essen, Essen, Germany

Correspondence:

Kira Schmidt, M.Sc.

Kira.schmidt@uni-due.de

ORCID-ID: 0000 0002 2308 509X

Clinic for Psychosomatic Medicine and Psychotherapy, LVR University Hospital Essen, University of Duisburg-Essen, Virchowstraße 174, 45147 Essen, Germany

Table 1

*Descriptive statistics of frequency bands (Hz) during first and sixth neurofeedback sessions over the mean 60 seconds of each training session*

| Outcome | |  | *N* | *M* | *SD (SE)* | *Min* | *Max* |
| --- | --- | --- | --- | --- | --- | --- | --- |
| Session 1 | |  |  |  |  |  |  |
|  | First Alpha Training | | 18 | .725 | .214 | .463 | 1.144 |
|  | Second Alpha Training | | 19 | .677 | .262 | .00 | 1.159 |
|  | Theta-Beta Training | | 18 | 2.065 | .522 | 1.390 | 3.198 |
| Session 6 | |  |  |  |  |  |  |
|  | First Alpha Training | | 18 | .833 | .351 | .416 | 1.56 |
|  | Second Alpha Training | | 18 | .829 | .288 | .392 | 1.526 |
|  | Theta-Beta Training | | 18 | 2.038 | .552 | 1.279 | 3.148 |

Table 2

*Wilcoxon signed rank tests of mean frequency bands (Hz) of first and sixth neurofeedback sessions*

|  | *Z* | *p* |
| --- | --- | --- |
| First Alpha Training session 1 – session 6 | -2.158 | *≤.05* |
| Second Alpha Training session 1 – session 6 | -2.534 | *≤.05* |
| Theta-Beta Training session 1 – session 6 | .000 | 1.000 |

Table 3

*Correlation between psychometric data (quality of life, self-efficacy) and mean frequency bands (Hz) of first and sixth neurofeedback sessions*

|  |  | Quality of Life | | | Self-Efficacy | | |
| --- | --- | --- | --- | --- | --- | --- | --- |
|  |  | *Rho* | *p* | *N* | *Rho* | *p* | *N* |
| Total | First Alpha Training session 1 – session 6 | -.466 | .093 | 14 | -.333 | .245 | 14 |
|  | Second Alpha Training session 1 – session 6 | -.257 | .375 | 14 | -.280 | .333 | 14 |
|  | Theta-Beta Training session 1 – session 6 | .252 | .405 | 13 | -.261 | .388 | 13 |
| < 55 | First Alpha Training session 1 – session 6 | -.549 | .159 | 8 | -.747* | *≤.05* | 8 |
|  | Second Alpha Training session 1 – session 6 | -.600 | .116 | 8 | -.301 | .468 | 8 |
|  | Theta-Beta Training session 1 – session 6 | .396 | .332 | 8 | -.084 | .843 | 8 |
| > 55 | First Alpha Training session 1 – session 6 | -.759 | .080 | 6 | .551 | .257 | 6 |
|  | Second Alpha Training session 1 – session 6 | .091 | .864 | 6 | -.232 | .658 | 6 |
|  | Theta-Beta Training session 1 – session 6 | .335 | .581 | 5 | -.205 | .741 | 5 |
